# Two God-Kings, Two Skulls: Artificial Cranial Deformation in Akhenaten of Egypt and Khingila of the Huns

**DOI:** 10.7759/cureus.36751

**Published:** 2023-03-27

**Authors:** Matthew D Turner

**Affiliations:** 1 Transitional Year, Madigan Army Medical Center, Lakewood, USA

**Keywords:** body modification, khingila, akhenaten, annular acd, huns, ancient egypt, medical history, artificial cranial deformation

## Abstract

Separated by half a continent and over 1800 years, Akhenaten, the “Heretic Pharaoh” of Egypt, and Khingila, “The God-King” of the Alchon Huns, had a great deal in common. Both rulers laid claim to divinity, labeling themselves as gods amongst men, and both are represented in their official imagery with unusually shaped skulls consistent in appearance with artificial cranial deformation (ACD) performed upon them soon after birth. This article compares the evidence - including the KV55 mummy, likely the remains of Akhenaten himself - between the two God-Kings and determines that Khingila almost certainly possessed an annular erect ACD consistent with the wider Hun culture. Akhenaten’s ACD in his state-sponsored imagery was likely solely an artistic choice meant to emphasize his oneness with the divine. In both men, their represented ACD was ultimately intended to solidify their power through two different avenues. For Khingila, it was to emphasize a common ethnic and cultural heritage with his subjects. For Akhenaten, it was meant to set the pharaoh even further apart from the rest of humanity.

## Introduction and background

Artificial cranial deformation (ACD), the molding of neonatal skulls into permanent shapes, has been used in a wide variety of human cultures from the dawn of prehistory [1] to the modern age [2]. The breadth of reasons for the use of ACD is as vast as the diversity of cultures and groups that have used it, but one of the most important is to distinguish individuals - either by confirming a group identity or to further set them apart [2].

ACD has often been seen as a mark of intelligence and leadership granting one the right to rule, so much so that the term "heads of state" may originate from the use of ACD in medieval European nobility [2]. This paper sets out to examine two such rulers who have often been depicted with ACD, both of whom used their unique appearances as a means to claim the title of "God-King". While both Khingila of the Huns and Akhenaten of Egypt was depicted with various forms of ACD in their official state-sponsored propaganda, this paper sets out to establish that both self-proclaimed "God-Kings" did so for very different reasons and that likely only Khingila truly had a form of ACD.

## Review

Artificial cranial deformation

ACD is one of the oldest practices of body modification in existence, with the earliest known examples being two Neanderthal specimens from approximately 45,000 years ago [1]. Only possible in infancy “when the cranium or more specifically the calvaria is malleable” [2], it has been practiced across an extraordinary range of cultures, including the Inca, the Chinook of the Pacific Northwest, 19^th^ century France, sub-Saharan Africa, and many others. Some researchers even theorize that the term “heads of state” comes from the practice of infant ACD among medieval European nobility [2].

ACD is done for a variety of motives - including ethnic and cultural identity [3], the distinction between classes [4], a desire to make children more obedient [2], and even for a perceived “enhancement of beauty, health, and intelligence” [2]. While it is no longer practiced on infants to a significant extent today outside a few scattered enclaves, it is fortunate that ACD only has a minor effect on the development of the facial cranium and mandibular development and has been noted to have no discernible impact on intelligence [5].

Throughout history, there have been a number of techniques to accomplish ACD in a newborn. The most widespread is the use of tightly-wrapped fabrics and bandages [2], possibly the technique used by prehistoric Neanderthals [1]. Other techniques include manual massages, the use of “cradle boards” by some Native Americans [4], and the special *bandeau *head wrap of medieval France [2].

ACD can be broadly grouped into two general categories. The first, annular ACD, produces a circumferential skull through constriction of the head with pressure bandages. The result is a “distinctive conical cranial vault” that can be further subdivided into oblique and erect forms [3]. The second type, tabular fronto-occipital ACD, is accomplished through both anterior and posterior compression that produces flattening at the front and back of the head, inducing a lateral expansion of the head [3]. Tabular fronto-occipital ACD requires the use of more specialized equipment than annular ACD, such as the “cradle-boards” historically used by the Chinook tribes of the Pacific Northwest [2].

Both of the rulers discussed in this article are represented in their official iconography with annular ACD. Akhenaten’s long, sloping skull [6] is more consistent with that of annular oblique ACD, while Khingila’s erect “steeple head” [7] is consistent with annular erect ACD. This raises the question: why were these self-proclaimed God-Kings portrayed with such an unusual head shape? Had they both undergone ACD in infancy or are there deeper factors at play? To fully determine the answer, we must examine the two men in question.

Akhenaten

From 1353 to 1336 BCE, Egypt was ruled by “the most controversial and enigmatic of pharaohs”, the architect of a radical vision that “if it had survived, would have changed not just the history of ancient Egypt but, perhaps, the very future of humanity” [6]. Originally named Amenhotep IV, the second son of Amenhotep III rose to the throne of Egypt by sheer chance [6] - or perhaps what he interpreted as divine intervention. Abandoning the previous 1500 years of Egyptian polytheism, the young Pharaoh embraced the monotheistic cult of Aten the Sun God, and renamed himself Akhenaten, “effective for the Aten” [6]. In so doing, he declared himself as a co-regent with the sole Sun God, the personification of divine power itself [6]. As his reign progressed and Akhenaten’s religious fervor grew, he enacted a brutal campaign against all that violated the teaching of Aten. Temples that had operated for centuries were shut down, priests deposed, and a vast campaign of “state-sponsored iconoclasm” swept the kingdom as workers destroyed all references to the old gods of Egypt - even the plural form of the word “god” was chiseled from monuments [6]. In such an environment, it was common for the king’s subjects to hurriedly change their own names from references to the old gods to worship of the new Sun God in order to survive the “ritual murder of [Egypt’s] most cherished hopes and beliefs” [6].

Although previous pharaohs had grasped at divinity - Amenhotep III, Akhenaten’s father, famously declared that he had been conceived when the god Amun-Re visited his mother in the palace one night [8] - Akhenaten’s claims soon surpassed even that of his father’s. He often rode in an electrum-plated chariot that magnificently reflected the sun’s rays, appearing “dazzling like the sun itself” to the small army of guards and courtiers that followed him [6]. He rewrote centuries of political dogma - where before the pharaohs had claimed to uphold *maat *(an ancient Egyptian philosophy that roughly corresponds with truth, balance, justice, and order), Akhenaten declared that he was *maat*. In the Sun God’s realm, “truth no longer had an existence independent of the king’s actions: it was, by definition, whatever he wanted it to be” [6]. Even the traditional iconography of Egypt became oddly warped under Akhenaten’s rule. The pharaoh decreed that all his statutory embody his oneness with the sole creator-god, and so the artifacts we have of Akhenaten are a bizarre mix of masculine and feminine attributes. Many of the details became “distorted… the head was unnaturally elongated with angular and attenuated features… a narrow upper torso, which contrasted with a distended belly and broad hips… plump legs ended in spindly calves” [6]. The effect was so disconcerting that many early Egyptologists of the eighteenth and nineteenth centuries were unsure whether Akhenaten had been a man or a woman [9].

The reign of Akhenaten ended in its seventeenth year with the pharaoh’s death, and his son Tutankhamun (commonly known to the modern world as King Tut) took the throne. Tutankhamun was unable to replicate the achievements of his father and died at a young age soon afterward. The royal line of Egypt’s Eighteenth Dynasty died with him, and Akhenaten’s successors quickly took steps to eliminate all traces of the “heretic” pharaoh and his strange religion [6]. They were remarkably successful; by the time of the classical historians such as Herodotus and Manetho, there appears to have been no living memory of Akhenaten, for he is not present in any of their histories of Egypt [9]. For millennia, Akhenaten and his god Aten were confined to “total oblivion”, purged from the royal lists, chiseled from the monuments, and forgotten by the world [9]. 

**Figure 1:**
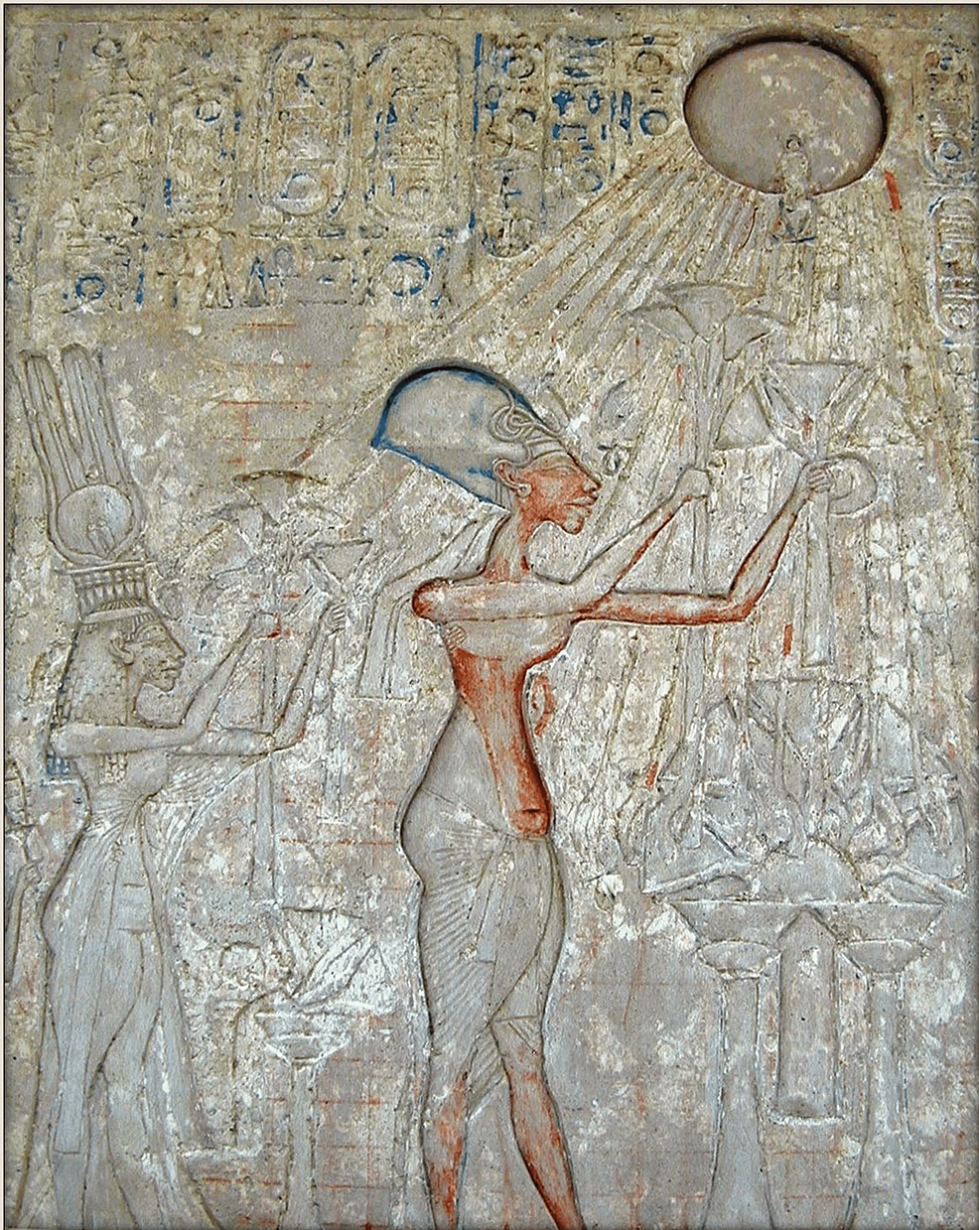
Akhenaten (center) and his family worshipping Aten, represented by the solar disc in the upper right corner. Note the unique structure of the pharaoh’s skull, highly reminiscent of annular oblique ACD, as well as his mix of male and female attributes. [13]

For our purposes, the unique iconography representing Akhenaten must be addressed. As previously established, the pharaoh’s unique representation portrayed him with both masculine and feminine features. In the past, researchers have theorized several etiologies for this, including a symbolic representation associating him with the creator Sun God [6], an aromatase excess syndrome [10], Marfan syndrome, Klinefelter syndrome, androgen insensitivity syndrome, Antley-Bixler syndrome [11]. The pharaoh’s head - with its odd emphasis on his unnaturally elongated skull [6] -particularly bears a close resemblance to that of annular oblique ACD, as shown in Figure 1. Akhenaten’s wife, Queen Nefertiti, famous to global audiences through the “Nefertiti bust”, displayed a similar skull shape in her official iconography [2]. Some researchers have suggested that Akhenaten had ACD performed on his infant daughters [12].

While evidence for ACD in ancient Egypt is virtually non-existent [4], the Eighteenth Dynasty may have been a notable exception. In the early 20^th^ century, researchers proposed that Akhenaten may have suffered from a form of hydrocephalus responsible for his appearance in the artwork of the time. His daughters, depicted in the same artistic style, may have had their heads artificially deformed in imitation of their divine father [14]. It likely would not have been out of character for Akhenaten to do such a thing: images of the royal family, including his six daughters, were routinely worshipped as “intermediaries” to the Sun God [6]. As the sole worshipper/co-regent that knew of Aten’s true plans for mankind [6], Akhenaten may have taken measures to literally shape his offspring into his own image.

While this is an intriguing theory, the evidence currently available to us suggests that it is highly unlikely. In 1907, a “small, single-chambered roughly-hewn rock tomb” was discovered by Edward R. Ayrton in The Valley of the Kings [15]. Within the tomb, designated KV55, excavators discovered a single mummy in a badly preserved condition. The poor quality of its tomb and its mummification immediately generated controversy among Egyptologists, many of whom initially declared, from its unique positioning (with the right arm straight down by the side and the left arm folded across its chest) and its pelvis that it was the remains of a woman within the royal household [15]. However, over the preceding decades, new evidence has come to light to suggest that the KV55 mummy is none other than Akhenaten. The mummy wore golden bands with the infamous pharaoh’s name inscribed upon them, and as more radiographic evidence came to light, was eventually determined to have been male. Genetic testing confirmed that he was the direct descendant of Queen Tiye and mummy CG 61074 (widely believed to be the remains of Amenhotep III) [16]. A 2010 genetic analysis of King Tutankhamun’s remains confirmed that the KV55 mummy was almost certainly his father [11]. Although the controversy over the KV55 mummy’s true identity remains [16], it appears that, after three millennia, Akhenaten has finally been rediscovered.

**Figure 2:**
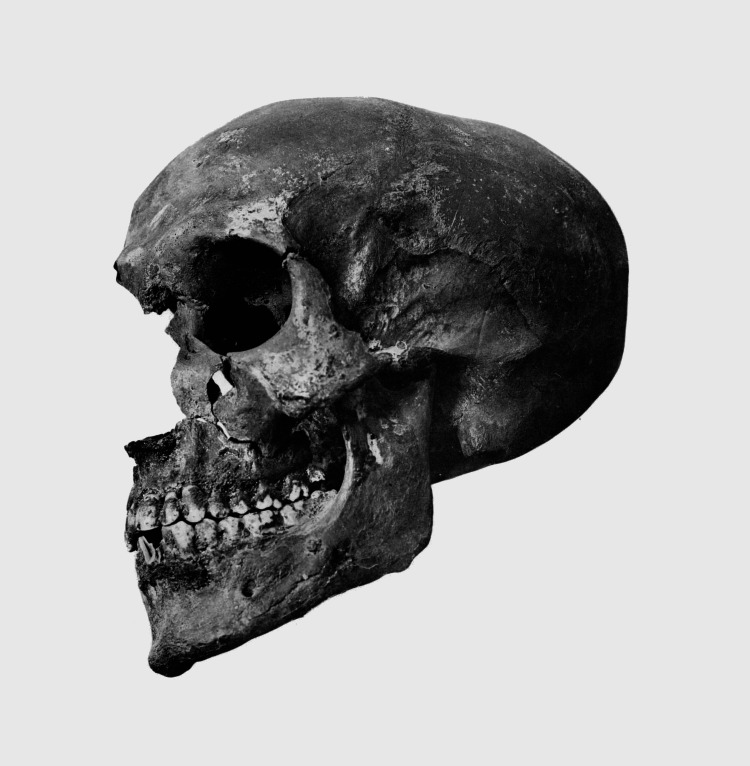
The KV55 skull, discovered in 1907 and widely considered to be Akhenaten’s. [18]

One of the most interesting aspects of the KV55 mummy is its skull, shown in Figure 2. Early Egyptologists maintained that the mummy’s skull displayed a “chronic hydrocephalus” with an enlarged and flattened cranium [17]. This description fits oddly well with the artistic representations of Akhenaten that have survived [6], suggesting that perhaps the god-king did have an ACD or some hitherto undiagnosed cranial abnormality. This assumption has since been called into question. However, in 1966, Harrison examined the skull of the KV55 mummy and concluded that it had an endocranial volume of approximately 1672 mL - megacephalic, but “nevertheless within the normal range for European adults” [17]. Furthermore, he concluded that the skull displayed no evidence of any significant abnormalities, with it being slightly brachycephalic - but still within “the normal range of variability of Eighteenth-dynasty skulls” [17]. Ultimately, his thorough analysis declares that the claim that “the skull is abnormally shaped such as to suggest artificial cranial deformation… cannot be accepted” [17]. Even the wider “bodily physique and proportions are also within normal limits” [17], corroborating the theory that Akhenaten’s iconography was purely symbolic and not intended to have been taken as a literal representation.

Modern CT imaging has supported Harrison’s findings. In a 2010 paper, researchers found that the KV55 skull had a cephalic index of 81.0 [11]. Tutankhamun’s skull, which some researchers have claimed resembles that of KV55’s [16], had a cephalic index of 83.9 [11]. In the wider literature, there is no consistent cutoff point to define brachycephalic via cephalic index - by some standards, the KV55 skull falls within a normal range [19]. Regardless of the specific definition used, the KV55 skull very clearly does not display the dolichocephalism that would be expected from Akhenaten’s imagery. Further investigation of Tutankhamun and the KV55 mummy also revealed no evidence of “gynecomastia, craniosynostoses, Antley-Bixler syndrome or deficiency in cytochrome P450 oxidoreductase, Marfan syndrome, or related disorders”, leading researchers to conclude that the representations of Akhenaten’s family were simply artistic choices “related to the religious reforms of Akhenaten… it is unlikely that either Tutankhamun or Akhenaten actually displayed a significantly bizarre or feminine physique” [11].

Although ACD was relatively common in the ancient Middle East, ACD within Egypt itself was a relative rarity. The oldest confirmed Egyptian skull to display ACD dates to the sixth century, some 1900 years after Akhenaten’s reign [4]. As we have just seen, the likely skulls of Akhenaten and his immediate successor display no evidence of ACD or any abnormality other than mild brachycephaly that was consistent within normal variation at the time [17]. It appears that there was no place for ACD in the Sun God’s kingdom.

Khingila

**Figure 3:**
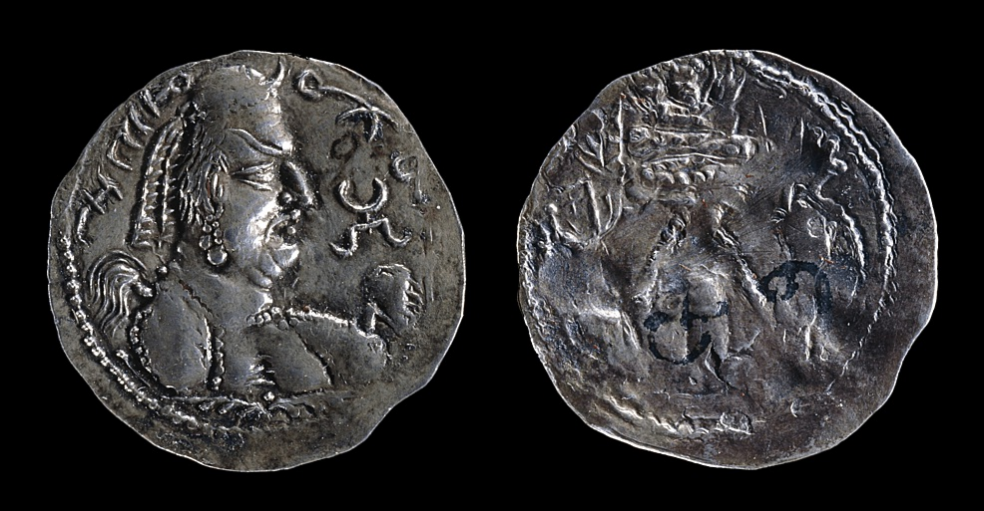
Khingila’s image engraved on a contemporary coin. Note the conical head shape consistent with annular erect ACD. This image was used with permission from the British Museum [23].

While a wealth of information and evidence has survived regarding Akhenaten - including his remains, if the theories about the KV55 mummy are true - the physical evidence of Khingila, who lived over 1800 years closer to the present day, is far more lacking. Despite his status as one of the early rulers of the Alchon Huns (also spelled Alkhan), and thus the ruler of a vast territory that included much of modern Northwest India [20], even the dates of his life (approximately 430-495 CE) are tenuous. One of the sole artifacts regarding the Hun king’s existence is a small statue of the Hindu god Ganesha that dates to the early sixth century and was found in Gardez in modern Afghanistan. The pedestal bears an inscription that references Khingila, the “illustrious” king that consecrated it [21]. Even with the limited information on the king, his visage has survived on a handful of coins that reference him as *Deva Shahi Khingila*, “God-King Khingila” [21], one of which can be seen in Figure 3. Even more notable than Khingila’s claim to divinity is his portrayal on the coins is the “steeple head” that the visage portrays. While it is not always visible - on several of the coins available to us today, the king alternates between wearing a diadem and a crown that obscure his skull deformity - the coins that do display it portray a head shape remarkably consistent with that of an annular ACD [22].

As a subgroup of the larger Hun culture, the Alchon Huns acted as a melting pot and crossroads between India, Iran, and Central Asia during much of the fifth and sixth centuries. Much like Khingila himself, they did not leave many written sources behind, and “coins and inscriptions are our main sources of information” [7]. Their coins, which often displayed Greek script, indicating a European influence, have been found over a vast geographical area, including much of modern India and Pakistan [20]. The Alchon Huns’ influence was not limited to economics alone. Supported by the vast steppes to the north, which “supplied them with an unlimited number of men and horses” and taking advantage of India’s political instability at the time, they swiftly carved out an empire south of the Hindu Kush [7]. There is evidence to suggest that they sent an emissary to the Chinese throne during Khingila’s reign, and within their armies, they appear to have possessed a contingent of war elephants that numbered in the thousands [20]. Khingila himself significantly extended the Alchon Huns’ territory through conquest [20], perhaps explaining why he claimed the title of “God-King” for himself [21]. One of their most distinguishing features in the archaeological record is the “steeple head” deformation that is seen in their coinage, of which Khingila is only one example of many [20]. Among the Alchon Huns, an elongated skull was a source of pride that they used to distinguish themselves from other ethnic groups [7]. As with Khingila, the representations of the “steeple head” Alchon Huns [20] are consistent in appearance with annular ACD.

The practice of annular ACD was extremely widespread among the greater Hun cultural group, of which the Alchon Huns were only a small offshoot [7]. As the Huns swiftly expanded across the Eurasian steppes, up to 80% of the steppe population began to “shape their heads in the same manner” [24]. As a means of allowing the conquered to imitate their conquerors, such widespread ACD was a response to the “new social and political situations that resulted from the Huns’ invasion” [24]. The vast majority of this ACD was annular, as the circular form of manipulating the neonatal skull is the easiest, only requiring the use of tight bands of fabric [24]. Many skeletal remains from this period still display the imprint of the tightening bandages. In cases where one bandage was used, the imprint can often be seen encircling the forehead and the occiput. When two bandages were used, the bandages typically were fixed ahead of and behind the coronal suture to cross at the porus acusticus externus [3]. During the Great Migration Period of the 5^th^ and 6^th^ centuries CE, the Huns brought annular ACD from the Eurasian steppes to central Europe. In Late Antiquity Bulgaria, a region under the Hun’s control during this period, skeletal remains from “almost all age groups”, from toddlers to the elderly, display evidence of annular ACD, something that was not present amongst the population before Hun tribes came to the area [3]. Populations in the Balkans especially came to adopt the practice of annular ACD, so much so that the practice “lost its specific ethnic content, making it difficult to associate this type of deformation with a specific group” [25]. The influence of the Huns was so great that their attempt to make themselves “discernible from the others in a crowd” [3] ironically led the conquered peoples of their vast empire to “emulate their prestigious Hun conquerors both culturally and physically” through ACD [25].

While the physical evidence for Khingila is far more lacking than Akhenaten, it is almost certain that his iconography - displaying him with an annular ACD - is a true representation of his appearance. Not only was the annular ACD exceptionally common amongst the broader Hun sphere of influence [25], it was widely celebrated by the Alchon Huns and a point of significant ethnic pride [7]. In this author’s opinion, it is difficult to imagine Khingila rising to the position where he could declare himself “God-King” without possessing the appearance that his subjects would expect [21]. Khingila’s annular ACD was likely similar to other practices of the time and accomplished through the use of one or two bandages that were tightly wrapped around his head as a neonate in a circumferential pattern to create a distinctive conical cranial vault [3].

## Conclusions

Ultimately, the difference between Akhenaten and Khingila comes down to the difference in their chosen artistic representations. This author theorizes that Akhenaten, as the prophet of a new religious faith, intended to set himself apart from the rest of humanity, and the bizarre ACD displayed in his official iconography was one of the ways the pharaoh used to make himself appear both divine and otherworldly. 

It appears that Khingila portrayed himself with ACD precisely because he wished to establish a rapport with his subjects. As a proud Alchon Hun, his ACD likely created a sense of ethnic unity and belonging. While his remains have never been found, he did likely undergo annular ACD in the traditional context of the Huns; it is highly doubtful that he could have risen to such a high position otherwise. In this author's opinion, it was not Khingila’s proclaimed divinity that gave him ACD; it was his ACD that gave him a claim to divinity.
